# Compreendendo a Ligação entre a Gordura Visceral e a Saúde do Coração

**DOI:** 10.36660/abc.20240288

**Published:** 2024-06-21

**Affiliations:** 1 Universidade do Porto Faculdade de Medicina Unidade de Investigação Cardiovascular Porto Portugal Universidade do Porto Faculdade de Medicina - Unidade de Investigação Cardiovascular, Porto – Portugal

**Keywords:** Variabilidade do Batimento Cardíaco, Tecido Adiposo Visceral

As doenças cardiovasculares (DCV) continuam a ser um problema de saúde global significativo, com a obesidade e as suas anomalias metabólicas associadas contribuindo substancialmente para o risco de DCV. Nos últimos anos, a investigação tem-se centrado cada vez mais no papel do tecido adiposo visceral (TAV) na saúde cardiovascular, reconhecendo a sua contribuição única para a disfunção metabólica e o risco cardiovascular.^1 , 2^

A variabilidade da frequência cardíaca (VFC) é uma medida da variação nos intervalos de tempo entre batimentos cardíacos consecutivos, refletindo o equilíbrio entre os sistemas nervosos simpático e parassimpático – e, portanto, servindo como um valioso indicador da função autonômica e da saúde cardiovascular. A redução da VFC está associada ao aumento do risco cardiovascular, tornando-se uma ferramenta potencialmente útil para avaliação e monitoramento de risco em diferentes cenários clínicos.^3 – 5^

Neste número da revista, Habib SS et al., estudaram a associação entre TAV, medida pela avaliação da gordura visceral (AGV), e VFC em uma coorte de 99 homens sauditas saudáveis.^6^ O objetivo da investigação foi testar a hipótese de que a VFC difere entre os participantes em função da categorização do TAV, e que os índices de TAV estão mais fortemente associados à VFC do que a massa gorda relativa em homens adultos saudáveis.

Apesar de algumas limitações importantes, nomeadamente o desenho transversal (limitando a capacidade de estabelecer causalidade) e a composição da amostra de homens saudáveis (limitando a generalização para outras populações), este interessante estudo oferece informações valiosas sobre a complexa interação entre a adiposidade e a função do sistema nervoso autónomo e talvez de relevância clínica para avaliação de risco e orientação de prevenção primária.

Uma das descobertas mais marcantes do estudo foi a associação significativa entre os parâmetros AGV e VFC, particularmente "raiz quadrada média das diferenças sucessivas" (RQMDS) e "desvio padrão dos intervalos RR normais" (DPIN), que são indicativos de modulação parassimpática e variabilidade global, respectivamente. Curiosamente, indivíduos com níveis mais elevados de gordura visceral apresentaram redução da VFC, sugerindo comprometimento da função autonômica. É importante ressaltar que essas associações permaneceram significativas mesmo após ajustes para possíveis fatores de confusão, como idade, IMC e pressão arterial, ressaltando a robustez dos resultados.

Além disso, o estudo destaca a sensibilidade dos parâmetros da VFC às alterações na gordura visceral, superando medidas tradicionais como percentual de gordura corporal e relação massa muscular/gordura visceral (MMGV). Isto ressalta a utilidade potencial das métricas de VFC como marcadores sensíveis para monitorar o estado autonômico cardíaco em resposta a intervenções que visam a redução da gordura visceral.

Este estudo representa um avanço significativo na nossa compreensão da relação entre a gordura visceral e a saúde do coração. Ao identificar a gordura visceral como um fator de risco modificável para a função autonómica prejudicada, os resultados sublinham a importância de intervenções específicas destinadas a reduzir a acumulação de gordura visceral para melhorar a saúde cardiovascular. Modificações no estilo de vida destinadas a reduzir a gordura visceral, tais como mudanças na dieta e aumento da atividade física, poderiam potencialmente melhorar a função autonômica e mitigar o risco cardiovascular.

Além disso, este estudo sublinha a importância da investigação em curso nesta área para elucidar os mecanismos subjacentes que ligam a acumulação de gordura visceral à disfunção autonómica. À medida que novas perspectivas para a avaliação do risco cardiovascular e intervenções personalizadas estão claramente abertas com estas linhas de investigação, estudos futuros podem explorar a eficácia de intervenções que visam a redução da gordura visceral na melhoria da VFC e dos resultados cardiovasculares.
