# Supplementary material for: Compreendendo a Ligação entre a Gordura Visceral e a Saúde do Coração
Source: Arq Bras Cardiol. 2024 Jun 21;121(5):e20240288. [Article in Portuguese] doi: 10.36660/abc.20240288 (PMC11216327; doi:10.36660/abc.20240288)
Supplement: Translation

# Translation: Understanding the Link between Visceral Fat and Heart Health

**DOI:** 10.36660/abc.20240288i

**Affiliations:** 1 Porto Portugal Universidade do Porto Faculdade de Medicina - Unidade de Investigação Cardiovascular, Porto – Portugal

**Keywords:** Heart Rate Variability, Visceral Adipose Tissue

Cardiovascular disease (CVD) remains a significant global health concern, with obesity and its associated metabolic abnormalities contributing substantially to CVD risk. In recent years, research has increasingly focused on the role of visceral adipose tissue (VAT) in cardiovascular health, recognizing its unique contribution to metabolic dysfunction and cardiovascular risk.^1 , 2^

Heart rate variability (HRV) is a measure of the variation in the time intervals between consecutive heartbeats, reflecting the balance between the sympathetic and parasympathetic nervous systems – and, therefore, serving as a valuable indicator of autonomic function and cardiovascular health. Reduced HRV is associated with increased cardiovascular risk, making it a potentially useful tool for risk assessment and monitoring in different clinical scenarios.^3 – 5^

In this number of the journal, Habib SS et al., studied the association between VAT, as measured by visceral fat rating (VFR), and HRV in a cohort of 99 Saudi healthy men.^6^The purpose of the investigation was to test the hypothesis that HRV differs between participants as a function of VAT categorization, and that indexes of VAT are more strongly associated with HRV than relative fat mass in healthy adult men.

Despite some important limitations, namely the cross-sectional design (limiting the ability to establish causality) and sample composition of healthy men (limiting the generalizability to other populations), this interesting study offers valuable insights into the complex interplay between adiposity and autonomic nervous system function and may be of clinical relevance for risk-assessment and primary prevention guidance.

One of the most striking findings of the study was the significant association between VFR and HRV parameters, particularly "root-mean-square of successive differences" (RMSSD) and "standard deviation of normal RR intervals" (SDNN), which are indicative of parasympathetic modulation and global variability, respectively. Interestingly, individuals with higher levels of visceral fat exhibited reduced HRV, suggesting impaired autonomic function. Importantly, these associations remained significant even after adjusting for potential confounders such as age, BMI, and blood pressure, underscoring the robustness of the findings.

Moreover, the study highlights the sensitivity of HRV parameters to changes in visceral fat, surpassing traditional measures such as body fat percentage and muscle mass to visceral fat ratio (MMVFR). This underscores the potential utility of HRV metrics as sensitive markers for monitoring cardiac-autonomic status in response to interventions targeting visceral fat reduction.

This study represents a significant step forward in our understanding of the relationship between visceral fat and heart health. By identifying visceral fat as a modifiable risk factor for impaired autonomic function, the findings underscore the importance of targeted interventions aimed at reducing visceral fat accumulation to improve cardiovascular health. Lifestyle modifications aimed at reducing visceral fat, such as dietary changes and increased physical activity, could potentially improve autonomic function, and mitigate cardiovascular risk.

Furthermore, this study underscores the importance of ongoing research in this area to elucidate the underlying mechanisms linking visceral fat accumulation to autonomic dysfunction. As new perspectives for the assessment of cardiovascular risk and tailored interventions are clearly open with these lines of research, future studies may explore the effectiveness of interventions targeting visceral fat reduction in improving HRV and cardiovascular outcomes.
